# Creating patient-specific anatomical models for 3D printing and AR/VR: a supplement for the 2018 Radiological Society of North America (RSNA) hands-on course

**DOI:** 10.1186/s41205-019-0054-y

**Published:** 2019-12-30

**Authors:** Nicole Wake, Amy E. Alexander, Andy M. Christensen, Peter C. Liacouras, Maureen Schickel, Todd Pietila, Jane Matsumoto

**Affiliations:** 1 Department of Radiology, Montefiore Medical Center, Albert Einstein College of Medicine, 111 East 210th Street, Bronx, NY 10467 USA; 2 Center for Advanced Imaging Innovation and Research (CAI2R) and Bernard and Irene Schwartz Center for Biomedical Imaging, Department of Radiology, NYU Langone Health, NYU School of Medicine, New York, NY USA; 3 Department of Radiology, Mayo Clinic, Rochester, MN USA; 4 Department of Radiology, The Ottawa Hospital, Ottawa, Canada; 5 3D Medical Applications Center, Walter Reed National Military Medical Center, Bethesda, MD USA; 6 Materialise USA, Plymouth, MI USA

**Keywords:** 3D printing, Virtual reality, Augmented reality

## Abstract

Advanced visualization of medical image data in the form of three-dimensional (3D) printing continues to expand in clinical settings and many hospitals have started to adapt 3D technologies to aid in patient care. It is imperative that radiologists and other medical professionals understand the multi-step process of converting medical imaging data to digital files. To educate health care professionals about the steps required to prepare DICOM data for 3D printing anatomical models, hands-on courses have been delivered at the Radiological Society of North America (RSNA) annual meeting since 2014. In this paper, a supplement to the RSNA 2018 hands-on 3D printing course, we review methods to create cranio-maxillofacial (CMF), orthopedic, and renal cancer models which can be 3D printed or visualized in augmented reality (AR) or virtual reality (VR).

## Introduction

Advanced medical image data visualization in the form of three-dimensional (3D) printing continues to expand in clinical settings. Many hospitals have started to adapt 3D technology to aid in patient care, for use in medical student education, and for research applications. 3D printing originated in the 1980s and encompasses various processes intended to generate a physical model from a digital file [1–3]. Virtual Reality (VR) uses a computer to simulate an alternate 3D environment and allows for user interaction within this space. Augmented Reality (AR), which overlays 3D content in the users real environment, is another method of advanced image visualization that has great potential to transform how physicians access medical imaging data. 3D printed models and AR/VR experiences are expected to provide improvements in the visualization of medical images as compared to viewing medical images on a two-dimensional screen [4].

At this time, digital imaging and communications in medicine (DICOM) files cannot be used directly for 3D printing anatomical models. In order to generate patient-specific models for 3D printing and AR/VR, anatomical structures are segmented from DICOM data and the generated structures are converted to virtual 3D models. Next, these files must be saved in a format that is recognized by the 3D printer or AR/VR device. The most common file-type for 3D printing is the stereolithography file format, which is also known as the Standard Tessellation Language or Standard Triangle Language (denoted by the file extension “.stl”) and the wavefront or object (.obj) file type, which has the ability to include material properties such as color and shading, is most widely used for AR/VR applications [5].

In order to efficiently create 3D printed anatomic models and to use them safely for medical purposes, radiologists and medical professionals must understand the process of converting medical imaging data to digital files. Therefore, to educate radiologists and other medical professionals about the steps required to prepare DICOM data for 3D printing, hands-on courses have been taught at the Radiological Society of North America (RSNA) annual meeting since 2014. Our initial medical 3D printing guide was published for the 2015 RSNA annual meeting [6]. Since then, other guides have been published [7, 8] and there remains great interest regarding the many applications of medical 3D printing.

The RSNA 3D Printing Special Interest Group (SIG) has provided published recommendations regarding medical 3D printing [9]. Recommendations have undergone voting during a SIG business meeting by the active membership [9], including a position statement reflecting the use of United States Food and Drug Administration (FDA) cleared software to translate medical images into formats amenable to 3D printing for all aspects of patient care, defined by the SIG as all interactions with healthcare professionals, or patients and their families, related to medical care [8]. This course is educational and does not promote any product. In keeping with SIG recommendations, for the purposes of education we primarily focus on FDA-cleared software for the design and fabrication of patient-specific 3D models. The examples presented in this course include craniomaxillofacial (CMF), orthopedic, and renal cases.

The software used to create “Diagnostic use” anatomical models is considered by the FDA as a class II medical device. At the time the course was delivered, Mimics inPrint (Materialise, Leuven, Belgium) was the only software product with FDA clearance to create 3D printed anatomic models for diagnostic use. While details regarding FDA clearance are beyond the scope of this article, Mimics inPrint was cleared for craniomaxillofacial, cardiovascular, and orthopedic applications [10]. Regarding updates and questions, readers are encouraged to visit the FDA website or consult with the FDA for matters specific to medical 3D printing in the United States [11].

Cranio-maxillofacial 3D printing dates back to the late 1980s [12–14]. It is used today for the management of complex head and neck surgery, craniofacial surgery, endoscopic sinus surgery, and orthognathic surgery helping to ensure the correct resection of margins and repositioning of segments [15–20]. 3D printing in orthopedics dates back to the late 1990s [21], with current applications including upper extremity trauma, deformity, and arthroplasty; foot and ankle surgery; spine surgery; hip and acetabulum surgery; hip and knee arthroplasty; and orthopedic oncology [22–26]. 3D printing soft tissue structures such as the kidneys is relatively new, dating back only a few years [27–29]. 3D printed kidney cancer models can influence pre-surgical planning decisions, which may allow for enhanced performance of minimally invasive organ-sparing procedures [30].

Advanced imaging technologies such as 3D printing, AR, and VR have rapidly been gaining momentum in the medical field. There are many applications of advanced 3D technologies in medicine including pre-operative planning, procedure rehearsal, educational tools for teaching, and patient communication. Herein we review methods to create CMF, orthopedic, and renal cancer models which can be 3D printed or visualized in AR/VR. The ultimate goal is to educate participants about the steps required to create 3D anatomical models suitable for 3D printing, AR, or VR from DICOM images.

## Workflow

In general, the steps required for 3D anatomical modeling from DICOM data include the steps shown in Table 1. If imaging is performed with the intent to create an anatomic 3D model, the image acquisition parameters should be optimized for quality [31]. However, this remains challenging considering that imaging studies are typically performed before a model is ordered. Factors to consider include spatial resolution (approximately 1 mm^3^), reconstruction kernel, multi-phase contrast, metal artifact reduction, and sequence parameters for magnetic resonance imaging (MRI). Repeat imaging solely for the purposes of producing a 3D model is often not advisable because it is not cost-efficient and will increase patient radiation dose if a computed tomography (CT) scan is performed.

**Table 1:** Stages of the anatomical modeling process

Step 1: Image Acquisition	
- Select imaging modality	
- Set appropriate protocols	
- Export data to independent image post-processing workstation	
Step 2: Image Post-Processing	
- Isolate tissues and organs of interest	
- Prepare files for data visualization method of choice	
- Save and transfer data in an appropriate format	
Step 3: 3D Visualization or Physical Reproduction	
- Create 3D computer model	
- Prepare model for AR, VR, or 3D printing	

**Fig. 1:**
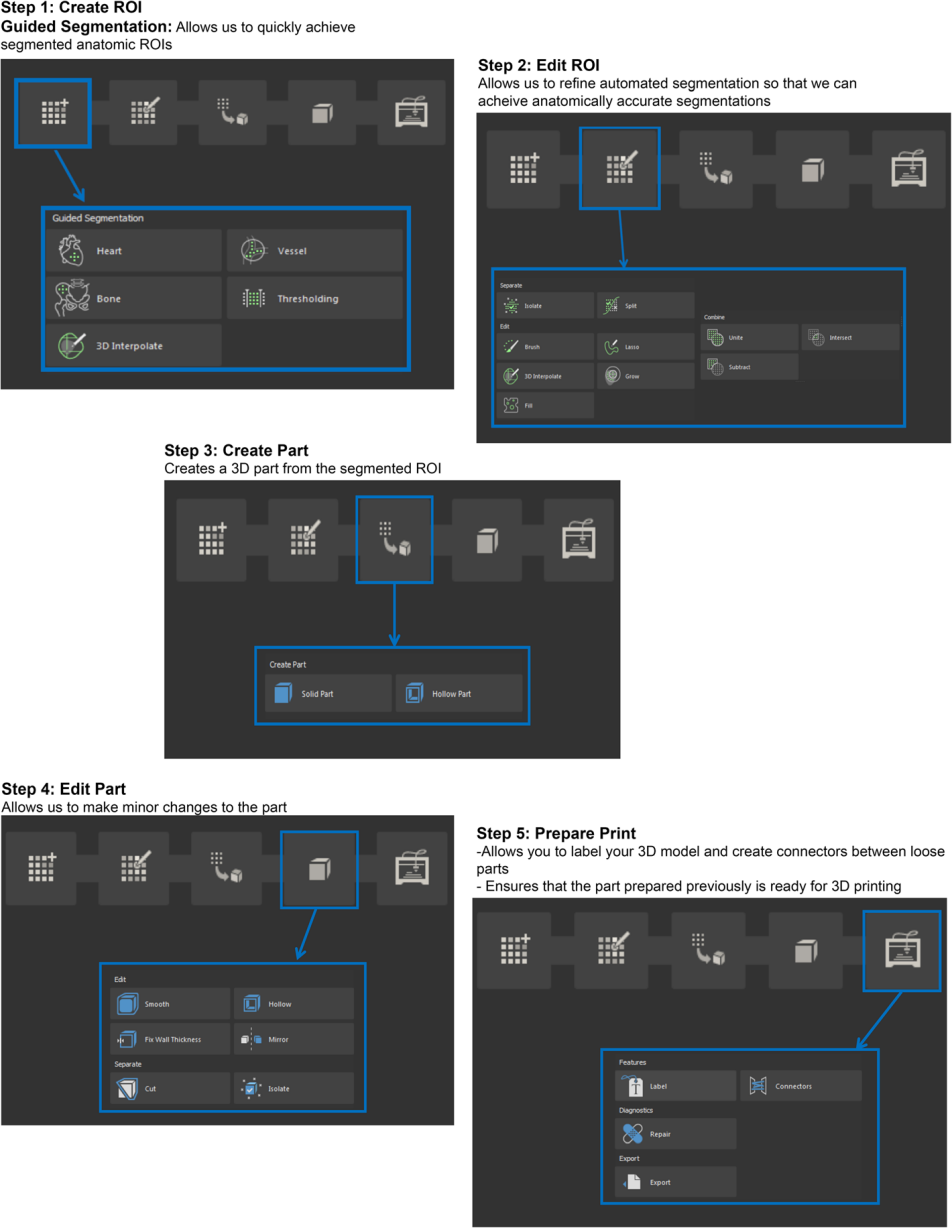
Mimics InPrint workflow steps including 1) Create ROI, 2) Edit ROI, 3) Add Part, 4) Edit Part, and 5) Prepare Print

Image segmentation and post-processing is performed with Mimics inPrint (Materialise NV, Leuven, Belgium). Mimics technology is widely used in academics, hospitals, and the medical device industry for 3D printing [32]. The Mimics inPrint software environment allows for a user friendly workflow to create anatomic regions of interest (ROIs) from the DICOM data and to convert the segmented imaging data to file types that can be used for 3D printing or AR/VR. The workflow consists of five steps including 1) Create ROI, 2) Edit ROI, 3) Add Part, 4) Edit Part, and 5) Prepare Print (Fig. 1). Here, each ROI is one segmented anatomical region and a part is the 3D representation of the segmented ROI. The main tools utilized to optimize how images are visualized in Mimics inPrint include zoom, pan, scrolling, zooming, one-click navigation, and threshold adjusting Table 2.

**Table 2:** Tools used in Mimics inPrint to optimize the visualization of images

Tool	Description	Shortcut 2D Viewport	Shortcut 3D Viewport
Zoom	Enlarges the view on the image slice or 3D ROIs/Parts	Right Mouse Button +drag	Scroll wheel -or-Ctrl + Right Mouse Button + drag
Pan	Planar movement of the image slice or 3D view	Middle Mouse Button +drag	
Scroll through image data	Jumps to the previous or next slice	Scroll wheel	-
Zoom in/out	Fills the viewport with the entire imaging slice or goes back to MPR view	Spacebar	
One-click navigation	Updates the slice position and 3D navigation location	Left Mouse Button -or-Shift + Left Mouse Button when a tool is active	
Adjust Threshold	Adjusts contrast window in images	Ctrl + Right Mouse Button +drag on image	

*This table has been modified from inPrint help manual

**Fig. 2:**
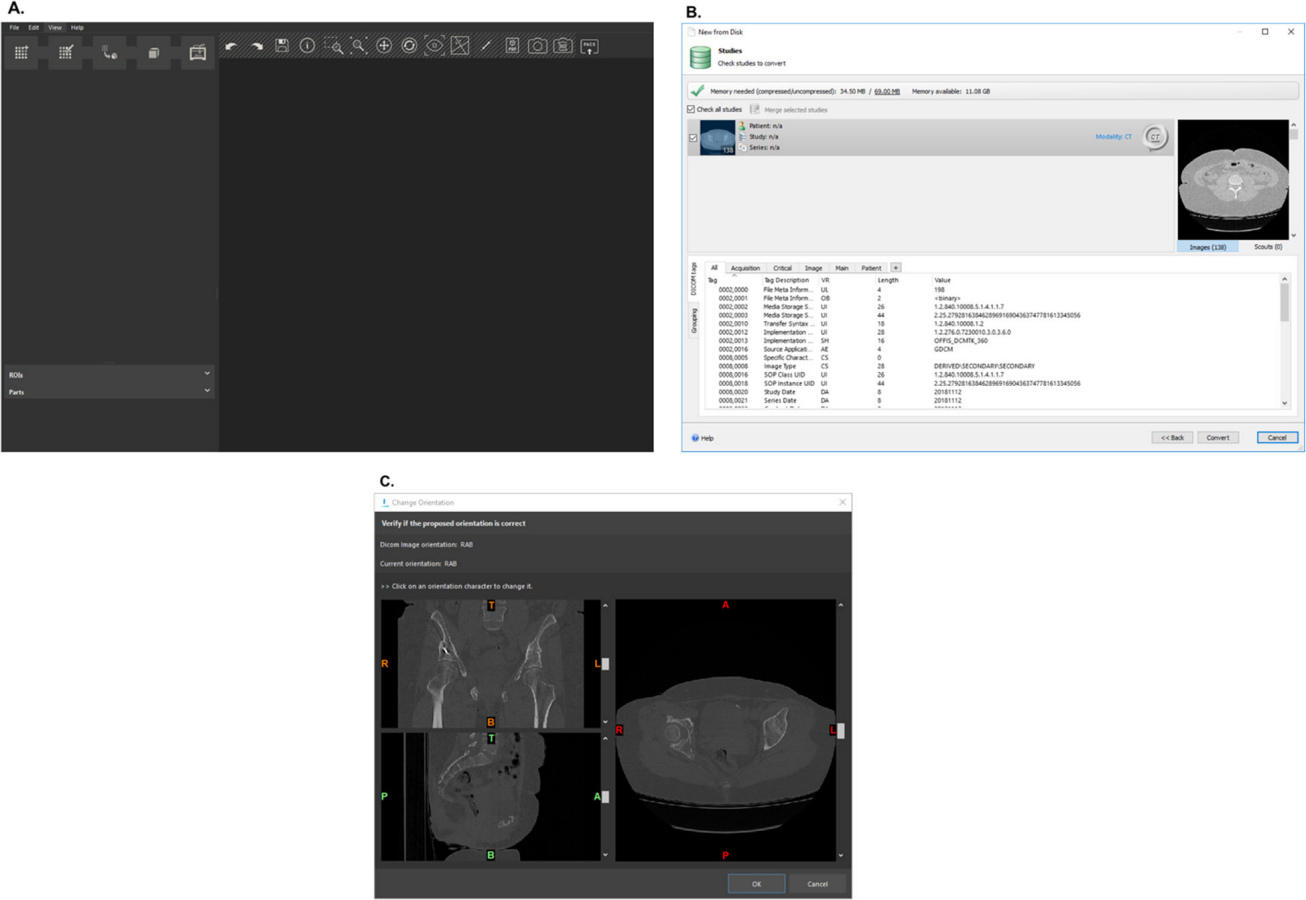
**a** Mimics inPrint software interface, **b** window to verify exam when loading DICOM images, and **c** window to verify orientation

When the software package is opened (Fig. 2a), a DICOM dataset can be loaded by following these steps: click *File ➔* select *New from Disk ➔* find folder where DICOMs are stored *➔* select the DICOM dataset, making sure that non-strict DICOM is checked *➔* select *Next* to import the images. A window will pop up with the selected study, which allows the study to be verified (patient name, date, number of images, etc.). Once the study is verified, make sure that the study is checked and click “*Convert”* (Fig. 2b). An orientation window will then appear (Fig. 2c) where the user can ensure the proper orientation is selected. This can be verified with the radiology report of the provider’s model request.

The principles of 3D printing in medicine are best understood through practical hands-on experiences covering a broad range of applications. Therefore, this guide will provide the foundational knowledge to broadly cover the segmentation of relevant anatomy on DICOM images followed by 3D printable model creation.

### Case 1: Pelvic Fracture

The pelvis is composed of paired hip bones that are connected in the front at the pubic symphysis and at the back by the sacrum. Each hip bone consists of three bones that fuse together during adolescence: the ilium, ischium, and pubis. The ilium, which resembles a butterfly wing, is the largest bone. Below the ilium is a ring-shaped structure made up of the ischium and pubis. The acetabulum, a cup-shaped socket that connects with the femoral head to form the hip joint, is the largest movable and weight-bearing joint in the human body.

Pelvic fractures may occur in any location; however due to the complex anatomy of the acetabulum and limited information from plain radiography, the acetabular fracture is the most challenging fracture to manage. 3D printed models can help surgeons to understand the volume, size, and orientation of the bone fragments, allowing them to determine the best reduction technique and surgical approach. 3D printed pelvic models can also lead to improved perioperative outcomes as compared to patients treated with conventional pre-operative preparation [33]. Mirror images of the opposite intact hemi-pelvis may also be created and can be used to pre-contour fixation plates and these have been reported to reduce surgical times [34, 35].

**Fig. 3:**
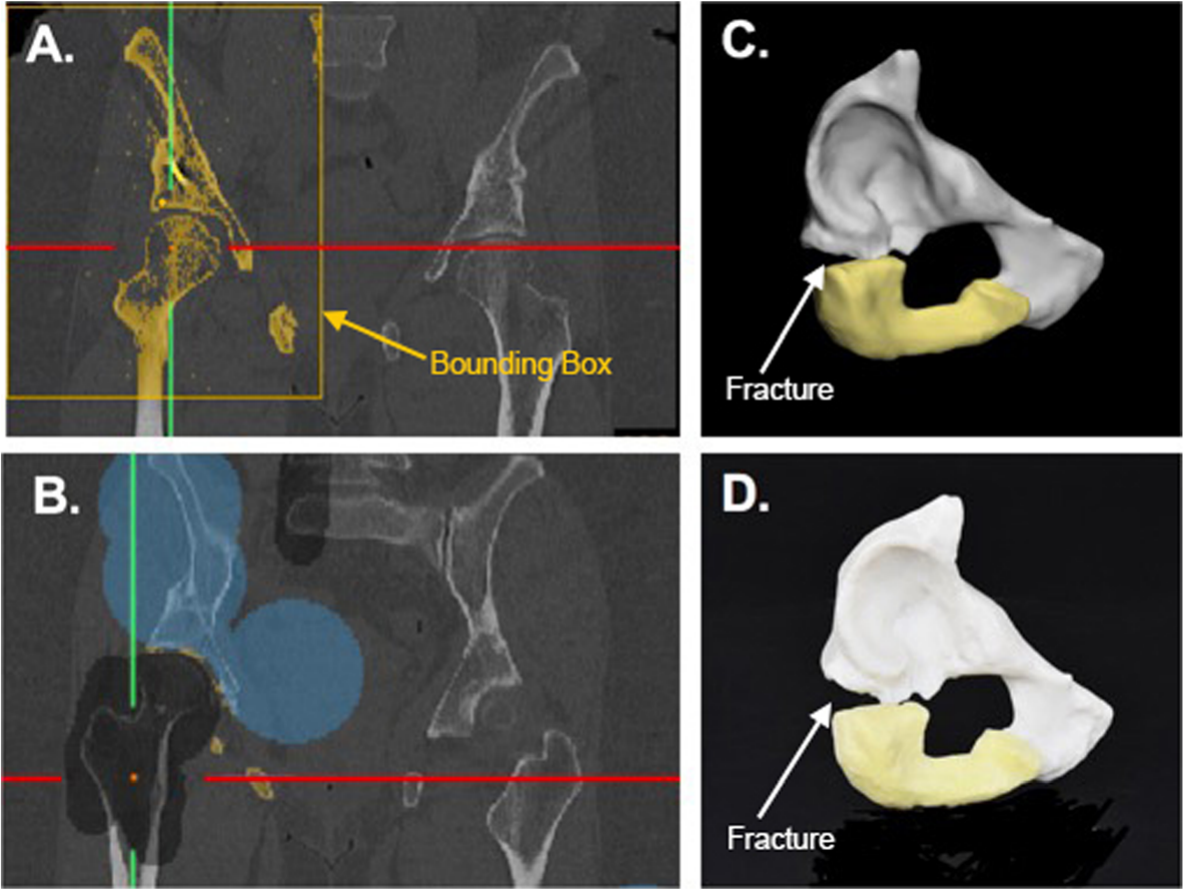
**a** Coronal CT image showing threhsolded right pelvic bones, showing similar colors for the pubis, ischium, and femur. **b** Coronal CT image showing splitting of the pelvis (blue) from the femur (black). **c** 3D computer model showing the pubis (white) and ischium (yellow). **d** Photograph of 3D printed model

To create a pelvic fracture model, the bony anatomy is segmented from CT DICOM data obtained with a 512 × 512 matrix and 0.781 mm pixel spacing. For CT images, a good threshold for bone segmentation is between 226 and 3071 Hounsfield Units (HU). Here, the pelvic fracture is on the right side, therefore the bounding box, a box that defines how much of each image is depicted in each window, can be cropped in the coronal, axial, or sagittal viewports to include only the right pelvis (Fig. 3a).

In order to separate the femur from the pelvis, the *“Split”* tool is utilized. Here, the foreground, the part that we want to keep, is highlighted in blue; and the background, the part that we want to eliminate, is highlighted in gray (Fig. 3b). Painted areas can be drawn in any 2D viewport and slice. After drawing the pelvis on at least 3 images, clicking the *“Validate”* button completes the bone segmentation leaving us with just the pelvis portion. Depending on the clinical request, the entire pelvis construct could be prepared for printing or the area could be reduced to just highlight the fractured section. Here we have only included the fractured part in the model. Also, the pubic bone and ischium were divided (Fig. 3c) so that they could be printed using material jetting technology (Stratasys J750, Eden Prairie, MN) in two diferent colors highlighting the fracture (Fig. 3d).

The contralateral side was segmented using the same methods described above and was mirrored onto the fractured side using the *“Mirror”* tool in order to help guide the surgical procedure. This plan is then exported in 3D portable document format (PDF) for visualization (Additional file 1).

### Case 2: Mandible Tumor

The mandible, the largest of the facial bones, is a single bone connected to the skull by the temporomandibular joint. Malignant tumors of the mandible constitute a diverse group of lesions [36]. 3D printing of mandibular tumors can improve comprehension of anatomy and with the production of cutting guides can enable fast, accurate mandibular reconstructions [37, 38].

In this case, a 52-year-old female presented with an ameloblastoma of the left ramus/mandible. Structures of interest to be included in the 3D printed model include the mandible, tumor, inferior alveolar nerve, and a floating wisdom tooth. The surgey will involve a full-thicknes resection of the mandible in the area of the tumor while attempting to salvage the nerve. Physical simulation of the resection will allow for pre-bending of a titanium reconstruction plate before surgery, potentially saving surgical time and making for a more aesthetic outcome for the patient.

Pre-operative CT images were obtained with the following imaging parameters: 512 × 512 matrix, 0.33 mm pixel spacing, 1 mm slice thickness, FC80 kernel, and 40 mA.

#### Mandible

Bone segmentation is performed by setting the threshold between 226 and 3071 HU. In this case the bounding box can be cropped in the multi-planar reformat (MPR) view to include only the mandible. Selecting “Keep Largest Region” will ensure that only the largest segment of bone is included.

#### Tumor

To segment the tumor, the *“3D interpolate”* tool is used in combination with the threshold operation to define the shape of the tumor. The brush tool is used to outline the boundaries of the tumor on diferent slices. The diameter of the brush can be changed using the slide bar or by holding control, left mouse clicking, and dragging. The mode can be changed from “*Draw (+)”* to *“Erase (−).”* Here, the minimum and maximum thresholds should be − 1024 and 365 respectively.

#### Nerves

The nerves can be delineated by manually contouring with 3D interpolation or spline creation (Mimics V22.0, Materialise, Leuven, Belgium).

#### Teeth

A preset threshold for *“Enamel (CT, adult)”* defined as 1553–2850 HU is selected. The bounding box is cropped so that it covers the lower teeth and roots. All the teeth are selected, and manual editing is performed with the *“Brush”* tool in erase mode or the *“Lasso”* tool in the 3D viewport to ensure that the teeth including the floating wisdom tooth are appropriately selected.

**Fig. 4:**
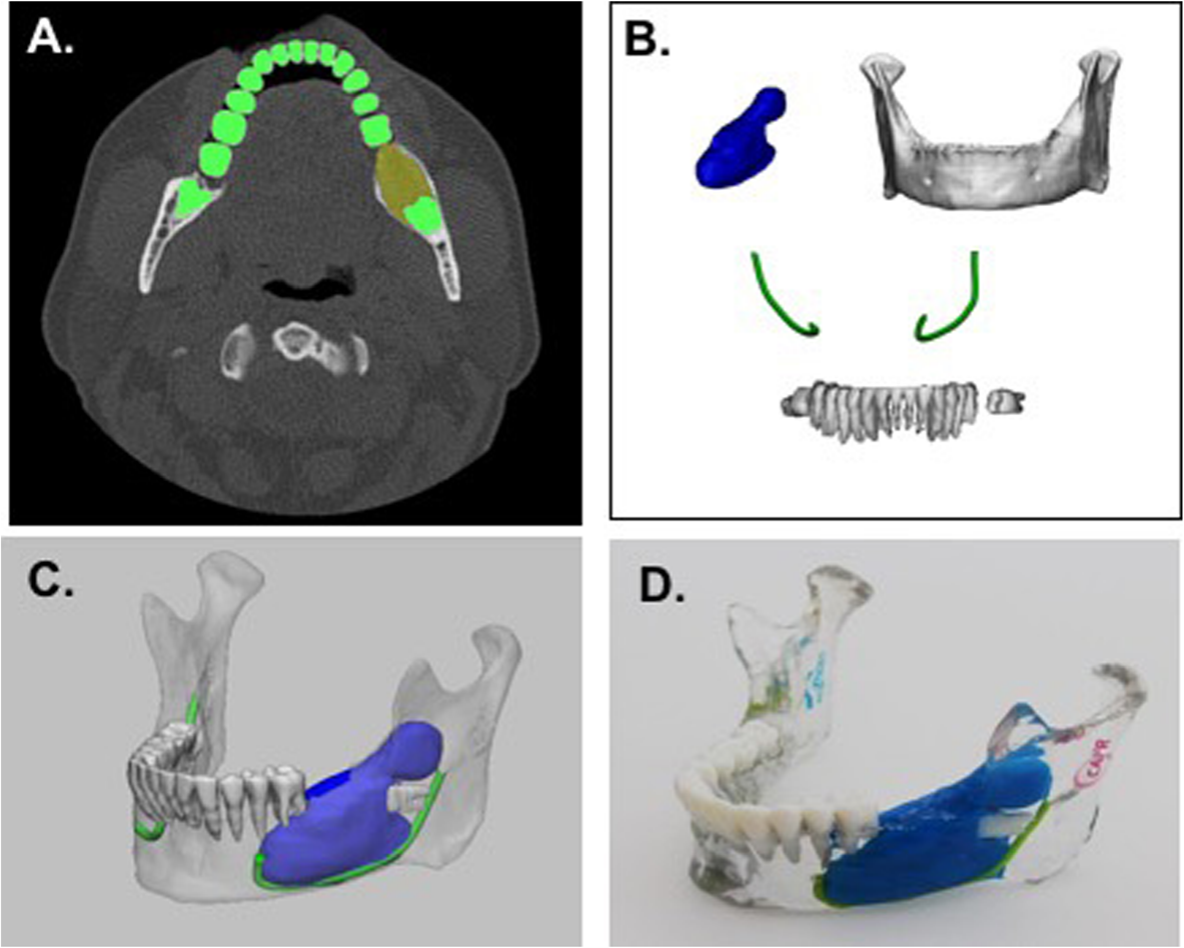
**a** Axial CT image showing segmentation of teeth (green) and tumor (yellow). **b** 3D anatomical regions of interest including the tumor (blue), mandible (white), teeth (white), and nerves (green). **c** 3D visualization of model including all anatomical parts. **d** 3D printed mandible tumor model including the mandible (clear), teeth (white), tumor (blue), and nerves (green)

The segmented anatomy (Fig. 4a) are converted to 3D parts (Fig. 4b) for better visualization and 3D printing and the 3D anatomy is viewed simultaneously (Fig. 4c). To best depict this anatomy, we chose to print using material jetting (Stratasys J750, Eden Prairie, MN) with the mandible transparent and the tumor and nerves in high presence colors such as blue and green. The total print time for this model was 9 h and 24 min using a high mix print setting; and the printed model is shown in Fig. 4d.

### Case 3: Kidney Tumor

Over the last 20 years, there has been an increase in the incidence of renal tumors, with renal cell carcinoma (RCC) accounting for approximately 3.5% of all malignancies [39, 40]. More complex kidney tumors are associated with longer operative times, warm ischemia times, and greater blood loss [41]. High kidney tumor complexity can also be correlated to the risk of major postoperative complications requiring a secondary intervention [42]. Patient-specific 3D renal tumor models may be used for partial nephrectomy or ablative therapy planning. Having a 3D model can help to assess tumor complexity, as well as the relationship of the tumor to major anatomic structures such as the renal vasculature and the renal collecting system [27, 29]. Models may help with improved education of the surgeons allowing for better surgical planning thus possibly reducing warm ischemia and operative times [30].

Here, we present the case of a 72-year-old male with an incidental right renal mass measuring 3.0 × 2.8 cm, Nephrometry score = 8 (moderate complexity). The patient decided to undergo robotic assisted partial nephrectomy and a 3D printed model was created to guide the surgical procedure. Pre-operative dual-energy CT images were obtained on a Somatom Force scanner (Siemens, Erlangen, Germany) with the following imaging parameters: 512 × 512 matrix, 0.69 mm pixel spacing, 0.6 mm slice thickness, 80kVp, Qr44d\4 convolution kernel. Isovue 370 contrast (Bracco Diagnotistics Inc., Monroe Township, NJ) was administered intravenously and arterial, venous, and delay phase images were obtained.

#### Kidney

To segment the kidney, the *“Threshold”* tool is used and the *“Kidney”* preset is selected. For this dataset an optimal threshold value is 60–1000 HU. The bounding box is cropped in the orthogonal 2D viewports, the *“Keep Largest Region”* box is selected, and the *“Validate”* button is clicked to proceed with the segmentation. Some of the tissue outside the kidney may be selected, so the *“Split”* tool is used to separate the kidney from the surrounding tissue. The kidney is marked as the foreground and the outside tissue is marked as the background. Once appropriately selected, the “*Validate”* button is clicked to move forward with the splitting function.

#### Tumor

The “*3D Interpolate”* tool is utilized to segment the tumor. Here, the *“Add”* option is utilized instead of the “*Threshold”* option. The tumor is outlined by drawing with a brush on at least 3 images. Once the tumor is nicely filled in on all of the views, the segmentation can be validated.

#### Artery

**Fig. 5:**
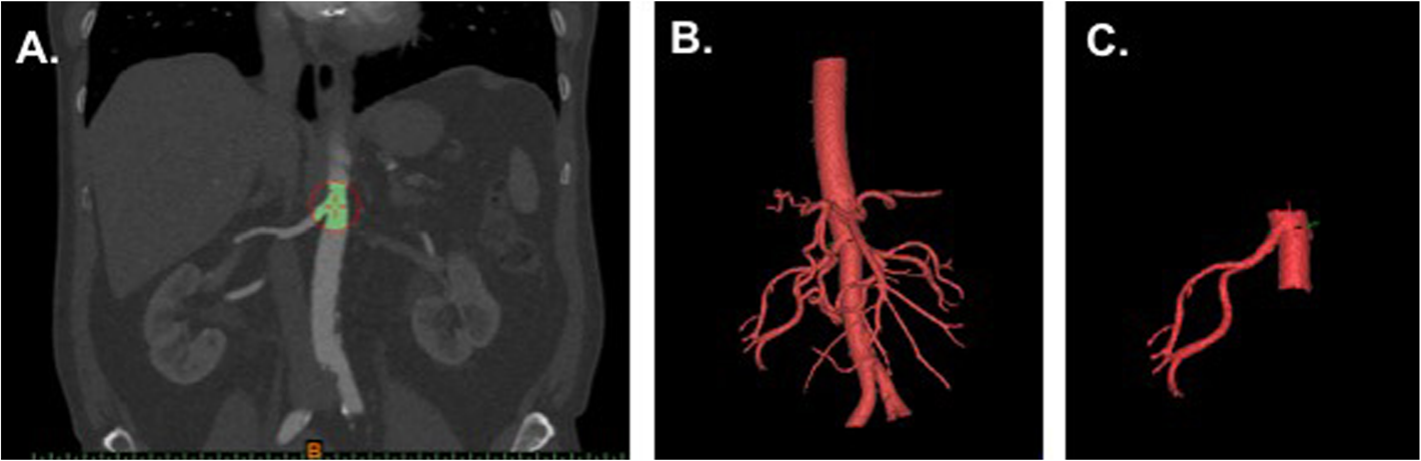
**a** Coronal CT image showing aorta and right renal artery selection. **b** 3D visualization of segmented arterial structures. **c** Remaining arterial region after trimming has been performed

Using the arterial phase, the *“Vessel”* tool is used to define the artery. Specifically, the *“Blood vessel (CT)”* preset is selected and the minimum threshold is adjusted to 300 HU. The renal artery is selected and the artery can be grown by left mouse clicking and dragging. There may be additional arteries included in the segmentation that we do not want to include in our final model. The extra vessels may be removed using the “*Lasso”* tool. Fig. 5 shows the arterial segmentation.

#### Vein and collecting system

The renal vein and renal collecting system are segmented using the venous and collecting phases respectively. As above, the *“Vessel”* tool is used to define each region of interest. The vein and collecting system are co-registered to the arterial phase using a point registration method and the segmented anatomical regions of interest are converted to 3D parts.

**Fig. 6:**
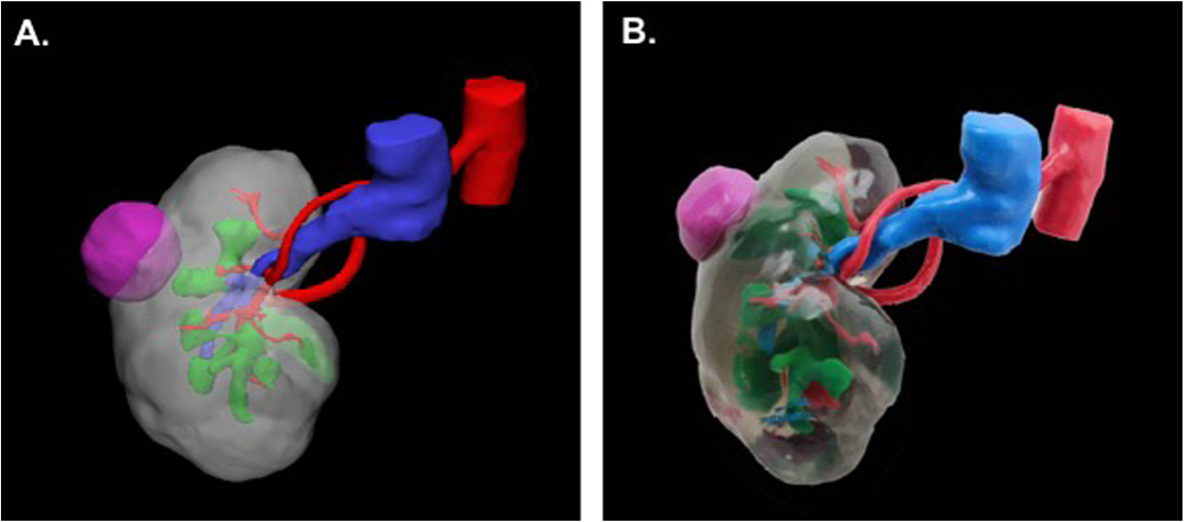
**a** 3D visualization of the kidney tumor model and **b** 3D printed model with the kidney (clear), tumor (purple), renal artery (red), renal vein (blue), and renal collecting system (green)

The segmented anatomy is combined (Fig. 6a) and printing is performed. Here we selected to print using material jetting with the kidney (clear), tumor (purple), renal arteries and aorta (red), renal vein and inferior vena cava (blue), and renal collecting system (green) (Fig. 6b).

**Fig. 7:**
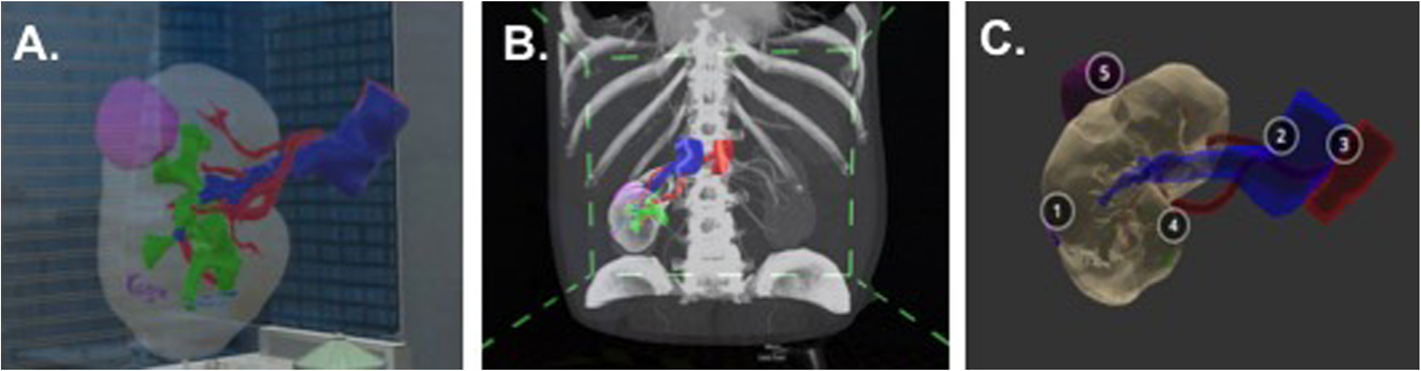
3D kidney tumor model visualized **a** in AR using the HoloLens AR headset (Microsoft, Redmond, WA), **b** in VR using Syglass software (Syglass, IstoVisio, Inc., Morgantown, WV) in combination with the Oculus Rift (Facebook, Menlo Park, CA), and **c** in VR using the Sketchfab app (Sketchfab, New York, NY) and a smartphone device. Each structure is numbered so that the unfamiliar user can easily identify each individual structure: 1 – kidney, 2 –vein, 3 – artery, 4 – collecting system, 5 – renal tumor

For all cases described above, an AR or VR model may be created from the segmented datasets. For preparation, each individual part can be exported in alias wavefront (.obj) format or each model including all of the parts can be exported in .vrml format. Models can be prepared in Unity, a cross-platform game engine (Unity Technologies, San Francisco, CA), for deployment in the AR headset [43] or can be visualized using a VR headset or cellular device (Fig. 7). The workflow for creating AR models in Unity has been previously described [44] and requires setting up a virtual camera and placing the 3D content a certain distance away for visualization.

## Conclusion

Converting DICOM data to printable formats is a complex process requiring multiple steps. This paper describes key steps to create 3D printed CMF, orthopedic, and renal models. Techniques described here may also be applied to other organs and anatomical regions of interest. The number of 3D printed and AR/VR models generated from DICOM images is growing exponentially at the point of care. It is essential that radiologists and other health care professionals understand this complex process.

## Supplementary information

**Additional file 1. Figure S1**: 3D PDF of the kidney tumor model. **Figure S2**: 3D PDF of the mandible tumor model. **Figure S3**: 3D PDF of the pelvic fracture model.

## Data Availability

The datasets used and/or analyzed during the current study are available from the corresponding author on reasonable request. We also plan to create a public link the the DICOM files, so readers can access the DICOM data used to create these models.

## Abbreviations

3D: Three-dimensional
AR: Augmented reality
CMF: Cranio-maxillofacial
CT: Computed tomography
DICOM: Digital imaging and communications in medicine
FDA: Food and Drug Administration
HU: Hounsfield unit
MPR: Multi-planar reformat
MRI: Magentic resonance imaging
OBJ: Wavefront object file
PDF: Portable document format
RCC: Renal cell carcinoma
ROI: Region of interest
RSNA: Radiological Society of North America
SIG: Special Interest Group
STL: Stereolithography, standard tesselation language, or standard triangle language
VR: Virtual reality
