# Supplementary material for: Creating patient-specific anatomical models for 3D printing and AR/VR: a supplement for the 2018 Radiological Society of North America (RSNA) hands-on course
Source: 3D Print Med. 2019 Dec 30;5:17. doi: 10.1186/s41205-019-0054-y (PMC6937827; doi:10.1186/s41205-019-0054-y)
Supplement: Supplementary file 1 — Additional file 1. Figure S1: 3D PDF of the kidney tumor model. Figure S2: 3D PDF of the mandible tumor model. Figure S3: 3D PDF of the pelvic fracture model. [file Mandible 3D PDF.pdf › Mandible 3D PDF.pdf]

materialise mimics
innovation suite

Rotate | Zoom | Pan | Full Screen Mode : CTRL + L
